# Multiple Biological Effects of an Iridoid Glucoside, Catalpol, and Its Underlying Molecular Mechanisms

**DOI:** 10.3390/biom10010032

**Published:** 2019-12-24

**Authors:** Subrat Kumar Bhattamisra, Kah Heng Yap, Vikram Rao, Hira Choudhury

**Affiliations:** 1 Department of Life Sciences, School of Pharmacy, International Medical University, Bukit Jalil, 57000 Kuala Lumpur, Malaysia; 2 School of Post graduate studies, International Medical University, Bukit Jalil, 57000 Kuala Lumpur, Malaysia; YAP.KAHHENG@student.imu.edu.my (K.H.Y.); VIKRAM.RAO@student.imu.edu.my (V.R.); 3 Department of Pharmaceutical Technology, School of Pharmacy, International Medical University, Bukit Jalil, 57000 Kuala Lumpur, Malaysia; HiraChoudhury@imu.edu.my

**Keywords:** catalpol, antidiabetic, anticancer, neuroprotection, antioxidant, anti-inflammation, cardiovascular protection

## Abstract

Catalpol, an iridoid glucoside, is widely distributed in many plant families and is primarily obtained from the root of *Rehmannia glutinosa* Libosch. *Rehmannia glutinosa* is a plant very commonly used in Chinese and Korean traditional medicine for various disorders, including diabetes mellitus, neuronal disorders, and inflammation. Catalpol has been studied extensively for its biological properties both in vitro and in vivo. This review aims to appraise the biological effects of catalpol and their underlying mechanisms. An extensive literature search was conducted using the keyword “Catalpol” in the public domains of Google scholar, PubMed, and Scifinder. Catalpol exhibits anti-diabetic, cardiovascular protective, neuroprotective, anticancer, hepatoprotective, anti-inflammatory, and anti-oxidant effects in experimental studies. Anti-inflammatory and antioxidant properties are mostly related for its biological effect. However, some specific mechanisms are also elucidated. Elevated serotonin and BDNF level by catalpol significantly protect against depression and neurodegeneration. Catalpol demonstrated an increased mitochondrial biogenesis and activation of PI3K/Akt pathway for insulin sensitizing effect. Further, its cardiovascular protective effect was linked to PI3K/Akt, apelin/APJ and Jak-Stat pathway. Catalpol produced a significant reduction in cell proliferation and an increase in apoptosis in different cancer conditions. Overall, catalpol demonstrated multiple biological effects due to its numerous mechanisms including anti-inflammatory and antioxidant effects.

## 1. Introduction

**Figure 1:**
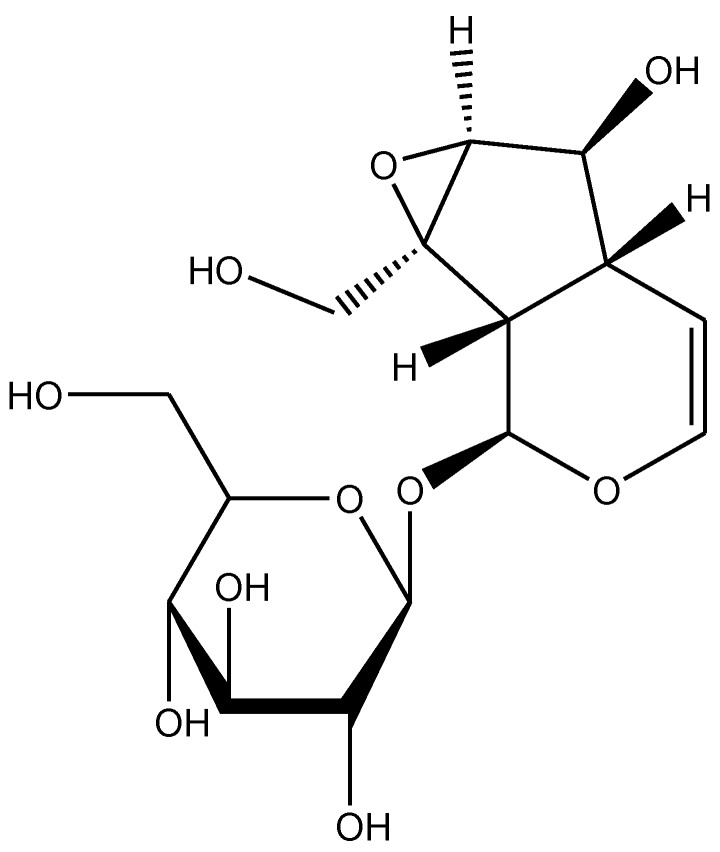
Chemical structure of catalpol.

*Rehmannia glutinosa* is a common traditional herbal medicine that is used for the treatment of aging-related diseases in Korea and China. This plant is extensively used as the name Di-Huang in Chinese traditional medicine for treating diabetes mellitus. Catalpol, an iridoid glucoside is the main active component derived from the root of this plant (Figure 1) [1]. Catalpol has been extensively investigated and shown to exert a wide variety of pharmacological effects; these include analgesic, sedative, liver protective, purgative, anti-inflammatory, anti-microbial, anti-tumor, and anti-apoptosis actions [2,3]. Catalpol was also isolated from the aqueous extracts of *Plantago lanceolate* (rib-grass, ribwort plantain) and leaves of *Buddleia* species [4]. Medicinal uses of *Plantago lanceolata* leaves are described in European Pharmacopoeia [5]. It is used as anticatarrhal for upper and lower respiratory tract, anti-inflammatory and antimicrobial effects [6]. Pharmacological properties of *Buddleia* species include anti-inflammatory, anti-oxidant, anti-hepatotoxic, antispasmodic, antibacterial, antifungal, hypotensive, and cytotoxic effects [7]. The other sources of catalpol are *Radix Scrophulariae* and *Lancea tibetica* [8]. This review focuses on the outcomes of research studies on catalpol conducted in both in vivo and in vitro experimental models and discussed the possible underlying mechanisms for its therapeutic potential.

## 2. Effects of Catalpol in Inflammatory and Oxidative Stress Conditions

Many studies have described the benefits of catalpol as an anti-inflammatory and anti-oxidative molecule. Some of its anti-inflammatory potential has been also discussed later sections, for example in the context of neurodegenerative, cardiovascular disorders, cancer and in hepatoprotective effect (Section 3, Section 5, Section 6, and Section 7.1). The anti-inflammatory potential of catalpol had also been reported in acute pancreatitis, hyperhomocysteinemia, diabetic complications, acute lung injury, multiple sclerosis, and various lipopolysaccharide (LPS)-induced inflammatory models [9,10,11,12]. The anti-inflammatory and antioxidant effects of catalpol are summarized in Table 1. The anti-inflammatory capability of catalpol was also observed in paw edema and broncho-alveolar lavage fluid of ovalbumin-induced mouse models [13,14]. A natural catalpol analogue, *6*-*O*-veratroyl catalpol significantly inhibited the expression of the pro-inflammatory cytokines IL-1β and TNF-α in THP-1 macrophages stimulated with phorbol-12-myristate-13-acetate (PMA) [15]. This effect appeared to be mediated by inhibition of ERK phosphorylation, possibly through down-regulation of PKC.

Asthma is a chronic and complex inflammatory disease characterized by airway inflammation, remodeling and hyperresponsiveness which is characterized by excessive mucus production leading to wheezing, coughing, and difficulty in breathing. Catalpol (5–10 mg/kg daily i.p.) alleviated inflammation of the airways and histopathological changes in the lungs of mice with ovalbumin-induced asthma; this was associated with the suppression of IL-4, IL-5, IL5Rα, and immunoglobulin E (IgE) [13]. Eosinophils have an important role in asthma and release toxic products at the sites of inflammation leading to airway remodeling and epithelial damage [18]. Catalpol reduced pulmonary eosinophil infiltration and suppressed OVA-induced elevation of the eosinophil chemokine and its receptor CCR3, suggesting that catalpol hindered eosinophil recruitment and activation in asthmatic mice [13]. This antiasthmatic effect of catalpol in ovalbumin-induced asthma in mice was confirmed by Zou and colleagues who showed that in addition to preventing symptoms, catalpol treatment significantly reduced the total number of inflammatory cells and eosinophils in broncheoalveolar fluid; this was accompanied by a marked reduction in immunoglobin E (IgE) and inflammatory cytokines (IL-1 and IL-4) and a marked increase in interferon-γ (IN-γ). Catalpol also produced a marked attenuation of TLR-4 expression and it was suggested that the antiasthmatic mechanism of catalpol may be linked to the suppression of the TLR-4 signaling pathway [14].

Over-production of ROS which leads to oxidative stress has been reported to be involved in several disease processes. The reduced nicotinamide adenine dinucleotide phosphate (NADPH) oxidase family, especially the NOX (1–5) genes are important sources of ROS. For example, oxidative stress has been found to play an essential role in the pathophysiology of atherosclerosis. Elevated NOX4 expression in atherosclerotic plaque has been found to promote oxidative stress and inflammation related to NF-kB activation [16]. As a result of these events, endothelial cells that express cell adhesion molecules such as intercellular cell adhesion molecule-1 (ICAM-1), monocyte chemotactic protein-1 (MCP-1) and vascular cell adhesion molecule-1 (VCAM-1) are promoted, causing increased formation of atherosclerotic plaque [19]. Catalpol increased superoxide dismutase (SOD) activity, the enzyme involved in cellular defense against oxidative stress, and suppressed ROS generation as shown by reduced NOX4 expression. The intercellular cell adhesion molecules that promote formation of atherosclerotic plaque were also down-regulated by catalpol treatment. Along with these benefits, catalpol also prevented apoptosis and attenuated autophagy deficiency. All of these effects were achieved via the activation of adenosine 5′ monophosphate-activated protein kinase (AMPK) [16]. The activation of AMPK leads to increased expression of SIRT1 and reduced deacetylation of p65 [16]. In an in vitro model, hydrogen peroxide (H_2_O_2_), a potent source of ROS that is able to overload the body’s natural antioxidant system causing damage and death of cells [20]. Overproduction of ROS down-regulates Bcl-2 while up-regulating Bax causing increased mitochondrial membrane permeability and triggering the release of mitochondrial cytochrome c to the cytosol. As a result, the caspase cascade is activated which in turn promotes apoptosis in response to death-inducing signals received from cell surface receptors or mitochondria [21]. Wang and colleagues demonstrated that catalpol was able to reverse the oxidative stress via the suppression of the caspase cascade in pheochromocytoma (PC12) cells [17]. In summary, catalpol shows promise in the treatment of disorders caused by excessive inflammation and oxidative stress as shown by its ability in reducing cellular damage and death.

## 3. Effect of Catalpol in Neurological Disorders

Catalpol was investigated for its effects in experimental models of depression, Alzheimer’s and Parkinson’s disease. Its effects are summarized in Table 2.

### 3.1. Depression

Catalpol has been investigated for its anti-depressant effect in several experimental models. When administered to mice for 14 days, catalpol (10 and 20 mg/kg, p.o.) like fluoxetine (10 mg/kg, p.o.) produced antidepressant-like effects; these effects were indicated by reduction in the duration of immobility in the forced swim and tail suspension tests, and by the attenuation of reserpine-induced ptosis, hypothermia and akinesia. Catalpol significantly increased 5-HT and 5-hydroxy-indoleacetic acid (5-HIAA) levels in mouse brain [22]. Catalpol, like major antidepressant drugs, could reverse the effects of reserpine which inhibits the vesicular uptake of monoamine neurotransmitters [22,30,31]. As catalpol only elevates the levels of 5-HT and 5-HIAA but not norepinephrine and dopamine, its anti-depressant-like effect may be mediated via the serotonergic pathway rather than the noradrenergic and dopaminergic systems [22].

Catalpol was also shown to ameliorate depression-like behavior in mice subjected to chronic unpredictable mild stress; this appeared to involve reconditioning the hypothalamic-pituitary-adrenal (HPA) axis, and enhancing the expression of tropomyosin-related kinase B (TrkB) and brain-derived neurotrophic factor (BDNF) [23]. These findings were in line with a previous study where hippocampal neurons subjected to a high corticosterone concentration were treated with an extract of *R. glutinosa*; here, catalpol protected the hippocampal neurons from glucocorticoid damage and from down-regulating the GCR-BDNF-NR2B-p-ERK-p-CREB-p-synapsin signal transduction pathway [32]. Catalpol further protected the forebrain neurons from neurodegeneration and improved memory through BDNF upregulation [24,25,26]. Depression has also been associated with immune system dysfunction. Elevated levels of pro-inflammatory cytokines such as interleukin-1β (IL-1β) and tumor necrosis factor-α (TNF-α) have been observed in patients suffering from depression. COX-2 mRNA and protein expression are increased in response to these pro-inflammatory cytokines [33]. COX-2 inhibitors were found to be neuroprotective several central nervous system (CNS)-related disorders such as epilepsy, Alzheimer’s disease, stroke and depression [34,35,36,37]. Catalpol, like fluoxetine, could also significantly reduce the expression of COX-2 and prostaglandin E2 (PGE2) in the frontal cortex and hippocampus of mice [23]. A double-blind, placebo-controlled, randomized study reported the therapeutic effects of the COX-2 inhibitor celecoxib in major depression [37], although there have been no such clinical studies using catalpol. Further, it was reported that inflammation leads to tryptophan depletion with consequent depletion of serotonin leading to depression in an animal model [37,38]. Overall, the data suggest that catalpol may exert antidepressant activity through increasing brain serotonin levels which could be linked to neuroprotective and anti-inflammatory effect. However, no clinical studies have been undertaken to determine if catalpol is effective in depression in the human.

### 3.2. Alzheimer’s Disease

The pathology of Alzheimer’s disease (AD) is characterized by the presence of neurofibrillary tangles and amyloid plaques in the brain, progressive loss of neurons and subsequent degradation of memory and cognition [39]. It is the most common age-related neurodegenerative disease and most drugs currently used in treating AD are palliative. The progressive cognitive deficits are mainly caused by the beta-amyloid peptide (Aβ) plaques in the brain resulting in gliosis causing inflammatory activation, neuritic and synapse disruption, neurofibrillary tangles, transmitter loss, and finally cell death [25]. Most current drugs for AD augment the cholinergic pathway in the brain through inhibition of acetylcholinesterase enzyme. However, catalpol was shown to enhance the activity of choline acetyltransferase (ChAT) and to reverse the effects of ChAT depletion, which is the cause for the shortening of neurite outgrowth and reduced interneuron connections [25]; additionally, treatment with catalpol significantly improved neuron survival rate and neurite outgrowth length [25]. Catalpol also increased BDNF content and expression in the forebrain neurons of mice with Aβ-induced neurodegeneration [25]. These mice showed improvements in memory deficits after treatment with catalpol, further supporting the beneficial effect of BDNF in AD; there was a significant positive correlation between BDNF concentration in the brain and cognitive ability [25]. In another mouse model, in which d-galactose was used to induce senescence, treatment with catalpol for two weeks produced significant neuroprotective effects. In this model, catalpol reversed the d-galactose-induced increase in acetylcholinesterase activity and reduction in ChAT in the mouse brain. The increases in pro-inflammatory cytokines (TNF-α and IL-1β) as well as in advanced glycation end products (AGEs) in the brains of senescent mice were also suppressed after treatment with catalpol [40]. In aged rats, catalpol increased hippocampal levels of the presynaptic proteins, synaptophysin and growth-associated protein (Gap-43) which are markers of neuroplasticity [26]. Wang et al. [27] confirmed the neuroprotective effects of catalpol via a novel non-amyloidogenic pathway. Swedish mutant amyloid precursor protein (APP) overexpressed N2a cells that enhances the production of Aβ were treated with catalpol. Aβ levels were found to reduce after treatment with catalpol however, APP, β-secretase, and γ-secretase complex expression levels were found to be unaffected by catalpol. This indicates that the inhibition of Aβ by catalpol is due to a non-amyloidogenic pathway and further proven by the increase in non-amyloidogenic process-related proteins such as α-secretase (ADAM10) and its proteolytic by-products, sAPPα and C83 via ERK/CREB signaling [27]. While experimental studies indicate the potential for catalpol as a treatment for AD through improving cholinergic pathway and inhibiting aging progression in experimental animal model, there is yet to be any clinical findings for substantiating this claim.

### 3.3. Parkinson’s Disease

Similar to AD, Parkinson’s disease (PD) is a chronic neurodegenerative disorder where current drugs are mostly palliative, with their effects gradually reducing overtime; they can also produce adverse effects such as dyskinesia [41]. PD results from the selective degeneration of dopaminergic neurons, with reduced availability of dopamine at the nerve terminals of fibers that project from the substantia nigra to the striatum [42]. In a mouse model of experimental PD produced by the administration of 1-methyl-4-phenyl-1,2,3,4-tetrahydropyridine (MPTP)/probenecid, treatment with catalpol for eight weeks improved locomotor ability and enhanced striatal dopamine, dopamine transporter levels and TH-positive neurons [28]. Furthermore, catalpol significantly increased protein levels of glial cell-derived neurotrophic factor (GDNF) [28]; increased GDNF expression has been hypothesized to restore dopaminergic neuron function [43].

Catalpol protected primary mesencephalic neurons in vitro from oxidative stress induced by the MTPT metabolite MPP^+^ [29]; oxidative stress is known to cause degeneration of dopaminergic neurons in PD and the neuroprotective effect of catalpol was proposed to be associated with its antioxidant activity [29]. The production of reactive oxygen species (ROS) together with the decrease in antioxidant enzymes level in the brain can result in enhanced oxidative stress and neuronal cell death [44] and catalpol was found to decrease the production of malondialdehyde (MDA) by cultured mesencephalic neurons, as well as to increase levels of the antioxidants glutathione peroxidase and superoxide dismutase (SOD) [29]. In this study, catalpol also significantly attenuated mitochondrial dysfunction, which has been reported to be involved in oxidative stress and dopaminergic cell death through reduced ATP synthesis.

**Figure 2:**
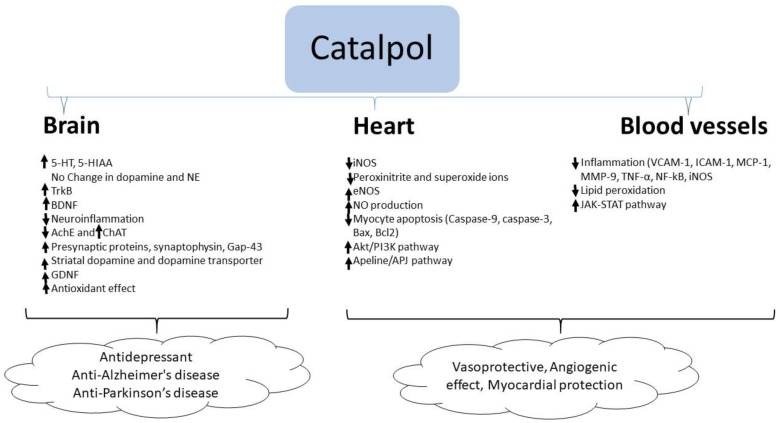
Molecular changes by catalpol for the protection of brain, heart, and blood vessels.

In summary, catalpol exhibits anti-inflammatory and anti-oxidant activities in the brain resulting in neuroprotection. Besides these effects, catalpol elevates the serotonin and BDNF level in the brain which supports its antidepressant effects. Similarly, catalpol increases acetylcholine concentration by stimulating choline acetyl transferase enzyme and BDNF level in the brain which correlates with its effect in improving experimental AD. In experimental models of PD, catalpol increased striatal dopamine concentration and GDNF expression suggesting its anti-PD effect (Figure 2).

## 4. Effects of Catalpol in Diabetes Mellitus

Diabetes mellitus remains a global threat and numerous studies have been undertaken on natural products as a potential source of new drugs for the treatment of this condition. The anti-hyperglycemic effect of catalpol was first investigated by Kitagawa and colleagues [8,45] afterwards by many researchers have studied its effect in experimental type 1 and type 2 diabetic models and its complications. The findings from different studies are summarized in Table 3. There have been no clinical studies to examine its effects in the human.

### 4.1. Type-1 Diabetes Mellitus

**Figure 3:**
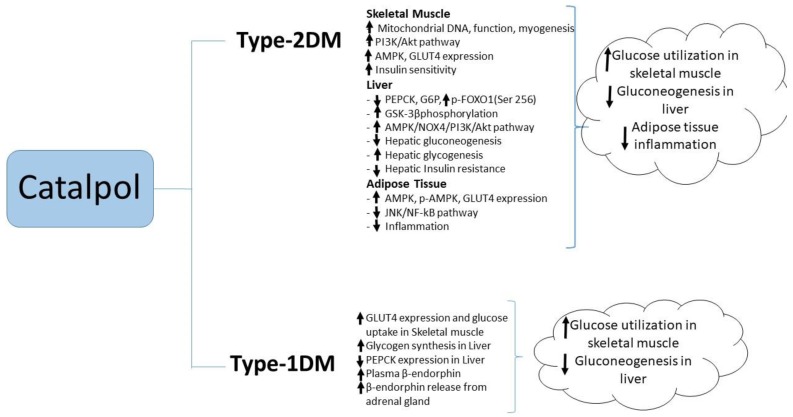
Molecular changes by catalpol for antidiabetic activity.

Most of the research studies used a chemically induced type-1 diabetic model where streptozotocin (STZ) was administered to rats in different doses [1,46,47,48]. Catalpol (50 or 100 mg/kg, p.o.), showed 59% and 72% reduction in blood glucose respectively after 4 weeks of treatment in STZ induced diabetes rat. The reduction in blood glucose is significant whereas, catalpol (10 mg/kg, p.o.) for four weeks did not reduce the blood glucose level significantly [47]. In another study in 2016, catalpol (10 mg/kg, i.p.) was administer to STZ injected C57 BL/6 mice for 14 days [49]. The blood glucose level in catalpol treated mice didn’t show any significant reduction as compared to diabetes control mice. Although the species and the route of administration are different in both the studies, catalpol at 10 mg/kg showed no blood glucose lowering effect considering as the sub therapeutic dose for type-1 diabetes model. Catalpol produced a dose-dependent (0.01–0.1 mg/kg, i.v.) antihyperglycemic effect in type 1 diabetes mellitus experimental model in an acute dose [46]; the reduction in blood glucose was maximal at 30 min after i.v. injection. Ex vivo studies showed catalpol (0.01–1.0 µM) to produce a concentration-dependent increase in radioactive glucose uptake in the isolated soleus muscle and to increase glycogen synthesis in hepatocytes isolated from these rats [46]. Unusually, and yet to be confirmed, the acute hypoglycemic effect in STZ rats appeared to be mediated by a catalpol-induced increase in the β-endorphin release from the adrenal glands, as evidenced by the increase in plasma β-endorphin, and such effects are prevented by opioid receptor antagonists naloxone or naloxonazine and by bilateral adrenalectomy [1]; moreover the acute hypoglycemic effect of catalpol was abolished in STZ-diabetic µ-receptor knockout mice. Three-day treatment of STZ-diabetic rats with catalpol significantly decreased hepatic PEPCK expression and increased GLUT4 expression in skeletal muscle; these effects were prevented by blockade of opioid μ-receptors [1]. The literature suggests that catalpol augments peripheral glucose utilization and attenuates hepatic gluconeogenesis in type-1 diabetic animals (Figure 3).

### 4.2. Type-2 Diabetes Mellitus

Catalpol was studied in several type-2 diabetes experimental models. In most of the studies, high fat diet/STZ induced type-2 diabetic model was used whereas db/db mice was also used in some studies. Li and colleagues showed catalpol (50–200 mg/kg p.o. for four weeks) to produce a dose-dependent reduction in fasting plasma glucose and a substantial reduction in serum total cholesterol (TC) and triglyceride (TG) concentrations in high-fat diet/STZ (HFD/STZ)-induced diabetic mice [50]. In this study, the hypoglycemic effect of catalpol was suggested to be mediated through improved muscle mitochondrial function as evidenced by increased mitochondrial ATP production, mtDNA copy number, mitochondrial membrane potential, and the increased expression of peroxisome proliferator-activated receptor gamma co-activator 1 (PGC1) α mRNA [50]. PGC-1α is the principal molecule that stimulates mitochondrial biogenesis and its expression is reduced in insulin-resistant and diabetic subjects [60]. Insulin-resistant subjects were reported to have fewer mitochondria in skeletal muscle, possibly due to low expression of PGC-1α and PGC-1β [61]. Thus, catalpol may produce its effects through increasing insulin sensitivity in skeletal muscle, although the exact mechanism remains unclear. A reduction in blood glucose accompanied by increased insulin sensitivity was demonstrated in db/db mice after oral administration of catalpol for eight weeks [51]. This was associated with activation of the PI3K/Akt pathway and augmented skeletal muscle myogenesis as evidenced by the increased expression level of myogenin (MyoG), myogenic differentiation (MyoD) and myosin heavy chain (MHC) proteins in skeletal muscle. Catalpol (10–100 µM) enhanced glucose uptake in C2C12 myoblasts via activation of PI3K/Akt pathway; and this activation was dependent on MyoD/MyoG-mediated myogenesis; associated with a marked elevation in the expression of MyoG and MyoD mRNA. These proteins are required for skeletal muscle development and myoblast differentiation during the process of myogenesis [51]. Thus, catalpol-induced increases in glucose uptake and insulin signaling in diabetic skeletal muscle could due to enhanced myogenesis [51].

Another study in db/db mice showed catalpol (40–160 mg/kg p.o. for 4 weeks) to significantly reduce the fasting blood glucose (FBG), glycated serum protein (GSP), TC, and TG concentrations with an improvement in glucose tolerance and reduced insulin resistance [52]. These metabolic changes were associated with increased GLUT-4 protein expression in skeletal muscle and adipose tissue and reduced expression of ACC and HMGCR in the liver [52], which might be explained by the increased expression of p-AMP-activated protein kinase (AMPK)α1/2 in the liver, adipose tissue and skeletal muscle observed in catalpol-treated mice [52]. Activation of AMPK in response to catalpol treatment (100–200 mg/kg daily p.o. for four weeks) was also observed in mice with type-2 diabetes induced by combined HFD/STZ. In this study, catalpol reduced blood glucose, ameliorated hepatic insulin resistance and reduced diabetes-associated hepatic injury and steatosis [53]; as well as activation of AMPK, catalpol treatment was associated with increased GSK3β phosphorylation, reduced glycogen synthase phosphorylation, stimulation of hepatic glycogen synthesis and inhibition of hepatic gluconeogenesis. The same group also showed that catalpol (10–180 µM) prevented insulin resistance induced by glucosamine in cultured HepG2 cells, in which catalpol inhibited the expression of PEPCK and G6pase and increased FOXO1 phosphorylation at Ser256; as in vivo, catalpol inhibited gluconeogenesis and increased glycogen synthesis in this in vitro model of insulin resistance. The in vitro effects of catalpol were blocked by pre-treatment with LY294002, a PI3K inhibitor, and by knockdown of NADPH oxidase type 4 (NOX4) or AMPK using short interfering RNA (siRNA). These findings suggested that the effects of catalpol in ameliorating hepatic insulin resistance were mediated through pathways involving AMPK/NOX4/PI3K/Akt [53].

Other studies have implicated the anti-inflammatory actions of catalpol in its ability to reduce blood glucose and ameliorate insulin resistance. In mice fed with a high fat diet, catalpol (100 mg/kg, p.o. for four weeks) reduced insulin resistance as evidenced by the reductions in fasting blood glucose and plasma insulin concentrations and the increased responsiveness to injected insulin [54]. These effects were accompanied by a reduction macrophage infiltration into adipose tissue, along with reduced adipose tissue expression of pro-inflammatory cytokines such as TNF-α, IL-6, and IL-1β and increased expression of anti-inflammatory markers such as IL-10. Reduced phosphorylation of IKKβ and JNK and reduced NF-kB p50 activation in adipose tissue from catalpol-treated mice suggested that the insulin-sensitizing effect of catalpol may be due to the attenuation of inflammation in adipose tissue through JNK and NF-kB signaling pathways [54]. Catalpol (5–50 mg/kg i.v. daily for two weeks) produced a dose-dependent reduction in blood in a rat model of STZ/high fat/high sugar-induced T2DM. In a maximally effective dose (50 mg/kg), catalpol also reduced blood concentrations of triglyceride and total cholesterol while significantly increasing blood concentrations of HDL cholesterol [55]. In this dose, catalpol also significantly increased the plasma concentrations of the antioxidant enzymes superoxide dismutase, catalase and glutathione peroxidase, while reducing plasma concentrations of malondialdehyde, a marker of oxidative stress [55]. Recently, Yap et al., reported that catalpol at (200 mg/kg, p.o.) showed significant reduction in FBG, HOMA_IR, plasma and liver triglyceride. The effect of catalpol was correlated with increased PPAR-γ gene and protein expression, glucokinase gene expression in the liver tissue, and further, the liver hepatocyte and glycogen content were reversed by catalpol in T2DM mice. This study suggested the role of catalpol in PPAR-γ expression that improves the insulin sensitivity in the liver [56]. A summary of catalpol’s effect against type-2 diabetes mellitus is illustrated in Figure 3.

### 4.3. Diabetic Complications

Diabetes is associated with several long-term macrovascular (coronary heart disease, stroke, and peripheral vascular disease) and microvascular complications (nephropathy, retinopathy and neuropathy) [62].

Two studies indicated a potential beneficial effect of catalpol on macrovascular complications of diabetes. Catalpol was found to protect the endothelium of the thoracic aorta in rats with diabetes induced by HFD/STZ. This was evidenced by a reduction in aortic levels of ROS and in serum concentrations of 8-iso-PGF2α and an increased serum concentration of NO and SOD. Moreover, catalpol reduced the expression of NADPH oxidase 4 (Nox4) and p22phox, major components of the free radical generating system, in the aorta from diabetic rats. Thus, the endothelial protective effect of catalpol was linked to a reduction in oxidative stress [57]. Catalpol was also shown to reduce neointimal hyperplasia provoked by balloon injury in the carotid artery of STZ-diabetic rats; this effect was associated with a down-regulation of monocyte chemoattractant protein-1 (MCP-1) expression in the injured artery [48].

Although there have been no studies on the effects of catalpol on diabetic retinopathy or peripheral neuropathy, several studies have demonstrated protective actions of catalpol against experimental diabetic nephropathy [49,58,59,63]. While it is difficult to separate beneficial effects on diabetic nephropathy from blood glucose-lowering effects, in at least one study [49], the protective effect of catalpol on the kidney was not associated with any hypoglycemic action. A number of mechanisms have been proposed for the beneficial effects of catalpol on the diabetic kidney. In a high fat-STZ model, 10-week administration of catalpol (30–120 mg/kg p.o.), in addition to reducing blood glucose, significantly reduced the elevated BUN and urinary protein excretion levels and attenuated kidney hypertrophy, the last effect being associated with a reduction in renal cortical extracellular matrix content [58]. This was accompanied by reductions in the renal cortical content of angiotensin II, TGF-β1, fibronectin (FN), connective tissue growth factor (CTGF), and collagen type IV (Col IV), along with reduced gene expression of CTGF and TGF-β1. TGF-β1 has an important role in diabetic nephropathy and is activated by angiotensin II [64]. Thus catalpol’s effect in reducing extracellular matrix accumulation in the kidney could be due to its ability to reduce the expression of TGF-β1, CTGF and Ang II [58]. In a study in STZ-diabetic mice, fourteen days of catalpol (10 mg/kg daily, p.o.) administration reduced urinary protein excretion, serum creatinine and BUN level [49]. The improvement in renal function was accompanied by increased levels of IGF-1 mRNA and IGF-1R phosphorylation, associated with down-regulation of growth factor receptor-bound protein 10 (Grb10), Grb10 being a negative regulator of IGF-1/IGF-1R signaling [49].

In db/db mice, sixteen-week administration of catalpol in the diet preserved renal function, as evidenced by a reduction in BUN, serum creatinine and proteinuria; the structural integrity of the kidney was maintained by catalpol, with increases in the density of glomerular podocyte processes, reduction in the size of podocyte gaps, a reduction in the glomerular basement membrane thickness, and reduced deposition of collagen fibers and glycogen [59]. Microarray analysis of the kidney suggested that catalpol could correct the deficit in the expression of genes responsible for lipid homeostasis and immune response resulting from leptin receptor deficiency in the db/db mouse [59].

Chronic diabetes can lead to central nervous system dysfunction manifest as diabetes-associated cognitive decline [65]; current therapies for diabetes mellitus do not completely prevent the development of this condition. One study in rats with STZ-diabetes showed that six-week administration of catalpol (10–100 mg/kg, p.o.) dose-dependently improved cognitive ability and reduced hippocampal neuronal damage; this was only partly attributable to correction of blood glucose, since at the lowest dose blood glucose in the diabetic rats was not modified by catalpol. The neuroprotective effects of catalpol were associated with its ability to prevent oxidative stress, as evidenced by the dose-dependent increase in hippocampal antioxidant enzymes (SOD, catalase and glutathione peroxidase) and reduction in malondialdehyde seen in catalpol-treated diabetic rats; AGE products were also markedly reduced by catalpol, which also produced a dose-dependent increase in hippocampal NGF content. [47]. Thus, long-term use of catalpol may benefit diabetic patients in ameliorating the central neuronal injury and deficiency in cognitive function [47].

## 5. Effect of Catalpol in Cardiovascular Disorders

Catalpol demonstrated marked cardiovascular protective effect in experimental models of atherosclerosis, myocardial infarct and ischemia. The effects of catalpol are summarized in Table 4 and Figure 2.

### 5.1. Myocardial Protection

Catalpol (5 mg/kg, i.p., 5 min before reperfusion) improved cardiac functions, reduced myocardial infarction, and reduced cardiomyocyte apoptosis and necrosis following 30 min of myocardial ischemia and 3 h of reperfusion (MI/R) in the rat. These effects of catalpol were accompanied by a significant attenuation of MI/R-induced iNOS expression, and peroxynitrite and superoxide anion production in the injured heart, as well as an elevation in the expression of Akt, an increased eNOS phosphorylation, and NO production, and an increase in the anti-oxidant capacity in the heart. All the beneficial effects of catalpol were completely blocked by wortmannin, a PI3K inhibitor, suggesting that catalpol’s cardioprotective effect is primarily due to enhancement of the Akt/ PI3K signaling pathway in cardiac tissue [66]. Catalpol was also found to be protective against isoproterenol-induced myocardial infarction in the rat [67,68,69], with catalpol treatment resulting in a significant improvement in cardiac function, as indicated by left ventricular end-systolic pressure (LVESP), left ventricular end-diastolic pressure (LVEDP) and left ventricular maximum rate of pressure development (LVdp/dt_max_) [68]. Catalpol also reduced the elevation in serum CK and showed an antioxidant effect, as evidenced by elevations in serum SOD and a reduction in serum malondialdehyde [67,69]. The study by Zeng and colleagues suggested that catalpol’s effects were mediated by activation of apelin and its receptor APJ, as evidenced by the enhancement of apelin and APJ expression levels in the plasma and myocardium [69]. As apelin can activate PI3K [74], this may explain the effect of wortmannin in preventing the effects of catalpol on myocardial/reperfusion injury [66]. Catalpol reduced cardiac myocyte apoptosis by reducing caspase-9 and caspase-3 activities; reduction in Bcl-2 like X protein (Bax) expression and increase in Bcl-2 expression levels towards normal [68]. This cardioprotective effect of catalpol may also involve endothelial progenitor cells mobilization and activation of the Notch1/Jagged1 pathway [69]. The mechanisms underlying the anti-apoptotic and anti-oxidative activity of catalpol were studied in vitro using glucose-deprived H9c2 embryonic rat cardiac cells [70]. This study suggested that catalpol alleviated the autophagy and mitophagy-related proteins in cardiac cells; the effects of catalpol were completely blocked by 3-methyladenine, an autophagy inhibitor and tamoxifen, an estrogen receptor blocker. Thus, anti-apoptotic and anti-oxidative activity of catalpol was linked to cell mitophagy and estrogen receptor modulation [70].

### 5.2. Vascular Protection

Catalpol (5 mg/kg daily p.o. for 12 weeks) reduced atherosclerotic lesions in rabbits fed a high cholesterol diet, this effect was accompanied by a marked reduction in circulating total cholesterol, triglycerides, and low-density lipoprotein cholesterol and an elevation in high-density lipid cholesterol [71]. Catalpol significantly reduced TNF-α, soluble VCAM-1, ICAM-1, IL-6, and MCP-1 in the serum and reduced the levels of VCAM-1, MCP-1, MMP-9, TNF-α, NF-κB, and iNOS protein in aortic arch tissue. Additionally, catalpol reduced lipid peroxidation levels, and elevated antioxidant activity [71]. Catalpol may have a beneficial effect in stroke in view of its ability to protect against middle cerebral artery occlusion in rats; in this model catalpol showed a significant vasoprotective and angiogenic effect through stimulation of the JAK-STAT pathway [72]. An in vitro study showed catalpol to protect human umbilical vein endothelial cells (HUVECs) against H_2_O_2_-induced apoptosis; here, catalpol pre-treatment significantly reduced intracellular ROS release, increased Bcl-2 expression, and decreased the expression of Bax. Catalpol increased Akt and Bad phosphorylation the effects were blocked by the PI3K inhibitors wortmannin or LY294002 [73].

## 6. Effect of Catalpol in Cancers

Cancer is the leading cause of death globally. In recent years, there has been increased interest in catalpol towards cancer cells. The effects of catalpol in various cancers are summarized in Table 5.

One of the most common cancers is gastric cancer; Wang and Zhan-Sheng showed catalpol (2.5–160 µM) to reduce proliferation and induce apoptosis in the human gastric cancer cell lines HGC-27 and MKN-45 [75]. Catalpol-treatment resulted in an increase in the proportion of cells in the G_0_/G_1_ phase while reducing the proportion in the S phase within the cell cycle; this as accompanied by an elevation of p53 and p27 expression and down-regulation of the expression of CDK4 and cyclin D1 genes. Catalpol significantly enhanced Bax expression levels, a pro-apoptotic protein, and increased caspase-3 activity, which is responsible for the cleavage of Poly (ADP-ribose) polymerase (PARP) resulting in the activation of the apoptotic pathway. In contrast, catalpol significantly reduced the expression of Bcl-2, an anti-apoptotic protein, thus indicating the mechanisms underlying catalpol-induced cancer cell apoptosis [75]. In vitro migration of gastric cancer cells was also reduced by catalpol; this was accompanied by down-regulation of several proteins that regulate cancer cell migration, including matrix metalloproteinase-2 (MMP-2), Ras homolog gene family, member A (RhoA), N-cadherin, Rho kinase-1 (ROCK1) and alpha-smooth muscle actin (α-SMA). These in vitro findings were supported by in vivo studies, in which catalpol dose-dependently reduced tumor volume and tumor weight in athymic nude mice bearing tumors produced by the injection of human gastric cells (HGC-27) [75].

In hepatocellular carcinoma (HCC) cells, catalpol significantly suppressed cell viability, colony growth, reduced the number of migrating/invading cells and increased apoptosis with an increase in the number of cells in the G_0_/G_1_ phase of the cell cycle. This anti-tumor activity of catalpol was correlated with up-regulation of miR-22-3p expression and down-regulation of MTA3 [83]. Using a number of solid tumor cell lines, catalpol was found to cause cell cycle arrest at the G_0_/G_1_ phase; this was associated with a decrease in cyclin D1 expression and the subsequent activation of the apoptotic pathway [76,84]. Catalpol is thought to act as a competitive inhibitor of DNA polymerase thus inhibiting DNA synthesis [84]. Pungitore and colleagues synthesized catalpol analogues with markedly increased potency in suppressing the proliferation of a panel of solid tumor cell lines, with concentrations producing 50% inhibition of cell growth in the range 1.8–4.8 μM, compared to catalpol, which had an IC_50_ of 48 μM [84].

In human non–small cell lung cancer (NSCLC) cells, catalpol showed significant inhibition of TGF-β1-induced epithelial mesenchymal transition (EMT) and inhibited TGF-β1-induced cell migration and invasion, as well as MMP-2 and MMP-9 expression. In addition, catalpol attenuated the Smad2/3 activation and NF-κB signaling pathways induced by TGF-β1 in A549 cells. The underlying mechanism suggested catalpol as a promising therapeutic agent for NSCLC treatment [77].

Several studies have also reported the beneficial effect of catalpol in experimental colorectal and colon cancers. Liu and colleagues showed catalpol (25–100 µg/mL) to inhibit the viability of human colorectal cancer cells; this was associated with an increased activity of caspase-3 and caspase-9 indicating induction of the apoptotic pathway. Catalpol also exhibited anti-proliferative activity associated with suppression of the activity of PI3K and Akt which are important proteins for regulating cell cycle and proliferation [78]; Akt is a downstream target protein of PI3K and the persistent activation of the PI3K/Akt signal transduction pathway has been associated with tumor development [85]. The anti-cancer activity of catalpol was suggested to be mediated via the down-regulation of the PI3K/Akt pathway, leading to increased expression of microRNA-200, thus promoting apoptosis and suppressing cancer cell proliferation [78]. Zhu and colleagues provided further evidence that catalpol could suppress growth, proliferation and invasion of colon cancer cells, this being achieved by inhibiting inflammation and tumor angiogenesis. Catalpol reduced inflammatory factors commonly found in colon cancer tumors such as IL-1β, IL-6, IL-8, COX-2, and iNOS [79]. Inflammation is one of the key mechanisms that cause progression of colon cancer [86,87]. IL-6 and IL-8 produced within the tumor microenvironment promote the growth and proliferation of colon cancer cells [88]. On the other hand, vascular endothelial growth factor (VEGF) is the most important growth factor that regulates angiogenesis. Along with VEGF, the receptor for VEGF binding (VEGFR2), hypoxia-inducible factor 1-alpha (HIF1-α) (a crucial regulator of the expression of angiogenesis-related factors), and basic fibroblast growth factor (bFGF) are essential in the angiogenesis process [88]. Normally, angiogenesis is kept in order by the balance of these pro-angiogenic factors and anti-angiogenic factors, such as angiostatin, canstatin, and endostatin. This balance shifts in tumors thus favoring pro-angiogenic factors. Catalpol significantly suppressed pro-angiogenic factors and restored the balance between pro and anti-angiogenic factors in colon cancer cells [78]. A placebo-controlled clinical trial in patients with advanced stage colon cancer who had recently undergone surgical resection showed that 12 weeks of treatment with catalpol (10 mg/kg of catalpol twice daily) resulted in a significant increase in overall and recurrence-free survival compared with the placebo up to 48 months after surgery [80]. Patients receiving catalpol had significantly lower serum levels of colon cancer biomarkers of carbohydrate antigen 19-9 (CA 19-9), carcinoembryonic antigen (CEA), MMP-2, and MMP-9 as compared to the placebo group. The reduction in these biomarkers may indicate that catalpol reduced metastases in colon cancer patients [89]. Adverse effects were significantly fewer that in patients receiving bevacizumab and, in contrast to bevacizumab, there were no fatal adverse effects [80]. This is the only published clinical trial on any effect of catalpol and provided very encouraging findings.

Catalpol has also shown potential for treating other types of cancers, including osteosarcoma, ovarian cancer and breast cancer. For example, catalpol (25–100 µg/mL) reduced proliferation and induced apoptosis in human MCF-7 breast cancer cells. This was associated with an activation of caspase-3, a reduction in MMP-16 activity, and an increased expression of the microRNA miR-146a [81]. Dysregulation of miRNAs has been reported to be involved in activating tumor-associated genes [90,91]. In particular, the expression of miR-146a was shown to reduce proliferation and to induce apoptosis in breast cancer cells, as well as to suppress CXCR4-mediated breast cancer migration [81,92,93]. While most studies have indicated a potential beneficial effect of catalpol in breast cancer, Hao and colleagues showed that in T47D cells, a model of progesterone-specific luminal A subtype of breast cancer, catalpol (10^−7^ M or 10^−6^ M) up-regulated pS2 mRNA expression, increased ER-α protein expression and significantly enhanced the proliferation in T47D cells; this effect was attenuated by the anti-estrogenic agent ICI182 780 (10^−8^ M). These authors concluded that catalpol has phytoestrogenic effects and stimulates estrogen receptors [94].

Catalpol (25–100 µg/mL) also reduced proliferation and induced apoptosis in the human ovarian cancer cell line OVCAR-3. This was associated with a decrease in MMP-2 levels and an increased expression of the microRNA miR-200. Overexpression of miR-200 suppressed MMP-2 levels, while the effects of catalpol on MMP-2 and on cell proliferation were reversed by anti-miR-200. Thus catalpol’s effect on these cells appeared to be mediated by increased expression of miR-200 with subsequent reduced expression of MMP-2 [95]. Catalpol similarly inhibited the proliferation and reduced the viability of two human osteosarcoma cell lines, MG63 and U2OS. These effects were attributed to the suppression of MMP-2 and Kras, a receptor for activated C-kinase 1 (RACK1) [82]. RACK1 plays an important role in the progression and metastasis of several cancers [96]. The activation of apoptosis in these osteosarcoma cells was associated with inhibited Bcl-2 expression and increased Bax protein levels, with enhanced cleavage of caspase-3/-9/-8 and PARP [82].

The above findings suggest that catalpol has great promise as a potential therapy for cancers given its ability to inhibit cancer progression and metastasis as well as inducing cancer cell death.

## 7. Other Biological Activities

Catalpol has also been shown to have hepatoprotective and ovary-protective effects. There are only a few studies supporting these claims and the effects are summarized in Table 6.

### 7.1. Hepatoprotective Activity

Pre-treatment with catalpol (2.5–10 mg/kg) significantly reduced liver damage and prolonged survival time in mice with acute liver injury produced by lipopolysaccharide (LPS)/d-galactosamine (d-gal) [97]; the reduced liver damage was evidenced by reductions in serum AST and ALT concentrations and by protection of the liver against the severe hemorrhagic necrosis, destruction of hepatic architecture, and massive infiltration of inflammatory cells. This protection was accompanied by reductions in MDA, MPO, and TNF-α and a reduction in activation of NF-κB activation. The expressions of Nrf2 and hemoxygenase-1 were up-regulated by catalpol treatment, suggesting that catalpol’s effects were due to the activation of the Nrf2 signaling pathway and inhibition of NF-κB signaling [97]. The hepatoprotective effect of catalpol was supported by the in vitro studies of Feng and colleagues, who showed that catalpol (2–250 µg/mL) protected human normal hepatocytes (L-02 cells) against triptolide (TP) induced hepatotoxicity. This protective effect of catalpol was relative to its anti-oxidative effect through by activation of Nrf2, NAD(P) H: quinine oxidoreductase 1 (NQO1), HO-1 expression and glutathione (GSH) activity [98]. Both these studies suggested that the hepatoprotective effect of catalpol was mediated by the nuclear factor erythroid-2-related factor-2 (Nrf2)/antioxidant response element (ARE) pathway [97,98].

Autophagy is a critical step in tissue damage that is regulated in a manner of homeostatic balance between degradation of damaged organelles, proteins and recycling materials. It is an important self-defense process during cellular damage [101]. It acts to suppress the pro-inflammatory cytokines and chemokines while facilitating the clearance of pathogenic microorganisms and damaged cells or organelles [102,103]. Autophagy plays an important role in most inflammatory-related disorders with one of them being hepatic fibrosis, which is due to chronic and excessive inflammation of the liver. Catalpol could protect the liver from carbon tetrachloride (CCl_4_)-induced damage as evidenced by attenuation of liver steatosis, necrosis and fibrotic septa. Not only did catalpol suppress pro-inflammatory factors like IL-1β, IL-6, IL-8, and TNF-α but also managed to promote autophagy in CCl4-induced liver fibrosis. Catalpol had promoted the expression of key biomarkers for autophagic flux, LC3-II and Beclin-1 indicating the increase in autophagic events upon treatment. Furthermore, the three key biomarkers of liver fibrosis, α-SMA, pro-collagen and fibronectin were also significantly reduced in catalpol treated CCl4-induced liver fibrotic rats [99].

### 7.2. Ovary Protective Effect

Administration of catalpol (1–5 mg/kg p.o. for four weeks) to aged female rats significantly reduced ovarian atrophy, while significantly increasing serum concentrations of estradiol and progesterone and decreasing the serum concentrations of FSH and LH levels. Moreover, the catalpol-treated rats showed the formation of new follicles in the ovary, a normalization of ovarian cellular organelle structures and an increase in secretory granules in the ovary. That the number of apoptotic ovarian granule cells was reduced in catalpol treated rats [100].

## 8. Pharmacokinetics of Catalpol

Most of the natural products are preferred to administer via oral route and therefore, the components experienced the issues of high gastrointestinal tract and hepatic fast pass metabolism and further oral bioavailability due to their physicochemical properties. Pharmacokinetic properties further influence the efficacy and safety of the products. Therefore, understanding of pharmacokinetic profile of the active compounds is crucial stage in the drug discovery and development. In catalpol development, pharmacokinetic consideration of catalpol is essential [104]. A number of HPLC, UPLC, HPTLC, LC–MS-MS, and micellar electrokinetic capillary chromatography methods have been developed for catalpol determination [105]. Tao et al., studies in vivo metabolic profiles of *R. glutinosa* extract in normal and chronic kidney disease (CKD) rats, using ultra-performance liquid chromatography/quadrupole time-of-flight mass spectrometry (UPLC-Q-TOF/MS), where they reported aglycone catalpol and followed by conjugation and hydrogenation correspondingly and acteoside was mainly conjugated by O-glucuronidation and O-sulphation [106]. In addition, in an in vitro study in human intestinal bacterial samples, Tao et al., separated and identified pure catalpol and its metabolites using UPLC-Q-TOF/MS method where they detected catalpol along with its four metabolites, catalpol aglycone, acetylated catalpol, hydroxylated catalpol, and nitrogen-containing catalpol aglycone [104].

A rapid and accurate and precise LC-electro spray ionization (ESI)-MS/MS method was developed and validated for catalpol quantification in rat plasma [107]. Catalpol was stable in rat plasma in room temperature for 4 h, upon three freeze thaw cycle, in auto-sampler at 4 °C for 12 h and long term stability conditions at −20 °C for 1 month. Further, the group determined oral pharmacokinetic parameters of catalpol at 50 mg/kg dose in Wistar rats using the developed and validated method. High rate of catalpol absorption was observed in their maximum plasma concentration (around 23 µg/mL) and time to reach maximum plasma concentration (around 1.3 h) value [107]. Faster elimination was evident from short elimination half-life around 1.212 ± 0.388 h. [107], which might be due to its hydrophilic nature. Another group also developed and validated a LC-MS/MS method, but with APCI (atmospheric pressure chemical ionization) instead of ESI for catalpol (m/z of 380/165) determination in rat plasma and cerebrospinal fluid (CSF) [108]. The group extended the long term stability to 68 days at −80 °C and found catalpol to be stable in a long term stability study. Afterwards, the method was applied to determine catalpol pharmacokinetic parameters after intravenous administration of catalpol at 6 mg/kg dose [108]. Similar findings in the other group, associated with quick elimination, was reported for catalpol. Elimination half-life was around 0.7 h in rat plasma, whereas elimination from CSF was comparatively slow with 1.5 h of elimination half-life reported. A maximum plasma concentration of around 23 µg/mL [107] and 0.6 µg/mL CSF concentration was reported. Brain penetration was reported with 5.8% area under the curve_(CSF)_ compare to area under the curve_(plasma)_ [108]. Xue et al. developed and validated a LC-ESI-MS/MS method for simultaneous determination of catalpol, ajugol, and aucubin in rat plasma [105]. Further, the method was validated based, for example, on linearity-, accuracy-, precision-, stability- parameters. Similarly, this group also confirmed catalpol stability in rat plasma under room temperature for 24 h, three freeze/thaw cycles, in auto-sampler at 4 °C for 24 h and also stable for three weeks under frozen conditions. The group investigated the pharmacokinetic parameters and tissue distribution of catalpol along with ajugol and aucubin after intravenous administration aucubin (2.0 mg/kg), ajugol (2.0 mg/kg), and catalpol (10.0 mg/kg). Quick elimination (elimination half-life of 0.984 ± 0.229 h) was reported for catalpol. Catalpol maximum concentration and elimination half-life was reported to be similar as previous single catalpol dosing [105,108]. Even faster elimination of catalpol also reported by Lu and colleagues [107]. Highest distribution of catalpol was detected in kidney, post dosing of 5 min, followed by other highly perfused tissue liver, such as heart and lung. Rapid brain distribution of catalpol was reported, which further strengthens the case for clinical use of catalpol in neurodegenerative diseases [105]. From the above study by Xu et al., it was found that there were no changes in catalpol pharmacokinetics parameters when it was administered along with other compounds [105].

After oral administration of drug pair extract prepared from the mixture of *Rehmannia glutinosa* and *Cornus officinalis* Sieb extract, at 4 mL/kg body weight in normal and CKD male Sprague–Dawley rats, pharmacokinetics parameters of morroniside, loganin, acteoside, and catalpol [109]. Simultaneous UPLC–MS method had been developed and validated for pharmacokinetic determination. Increased exposure and maximum plasma concentration was reported for catalpol along with other compounds in CKD rat compare to normal rats, which may be due to multiple physiologic changes in renal impairment model. Even metabolism also gets affected in renal impairment patients. In rats, intestinal total CYP levels reduced in CKD model, which further might result in enhance of oral bioavailability of many drugs [110,111]. Tao et al. reported metabolism of catalpol in intestinal microflora [104], which might be reduce in CKD model, therefore one of the reason for enhanced exposure and maximum plasma concentration of catalpol. Even in renal failure condition, reduced activity of some major efflux transporters was reported, which further can affect the disposition of drugs [112,113]. However, the reason behind the modification in pharmacokinetics behavior of catalpol along with other components in CKD rat model need to be further investigated [109].

Other team investigated pharmacokinetic of catalpol and puerain after intragastric administration of puerain and catalpol nanocrystal suspension to rats. Plasma elimination half-life of catalpol was reported to be 1.70 ± 0.4 h, whereas elimination half-life in brain homogenate increased to 3.88 ± 1.09 h. The plasma exposure of catalpol was reported to be 32.13 ± 5.29 mg·h/L and that in brain homogenate was 0.54 ± 0.16 mg·h/L [114]. Brain penetration was reported with 5.8% area under the curve_(CSF)_ compare to area under the curve_(plasma)_ by Wang and team [108], whereas in this study, brain penetration was reported with 1.5% area under the curve _(brain homogenate)_ compared to area under the curve_(plasma)_ [114]. The route of administration might affect brain penetration.

It can be concluded from the above review on metabolism and pharmacokinetic data of catalpol whether administered alone or with other compounds in normal experimental animals via different route of administration, catalpol has been shown faster elimination, which might be due to its hydrophilic nature. Further pharmacokinetics in CKD model, it has been found to alter pharmacokinetics parameters of catalpol, which might be due to alteration in metabolism and further enhanced absorption and slower elimination, however need investigation for further confirmation. The pharmacokinetic data of catalpol is illustrated in Table 7.

## 9. Conclusions

In addition to anti-oxidant and anti-inflammatory activity catalpol has shown significant neuroprotective effect against experimental Alzheimer’s disease and Parkinson’s disease; this could be related to the attenuation of neuroinflammation, free radical scavenging, modulation of neurotransmitter release and improvements in neuronal function. Catalpol has demonstrated potential glucose-lowering effect in experimental type-1 and type-2 diabetes mellitus; these effects may be related to improved glucose utilization in insulin-sensitive tissues and improved mitochondrial biogenesis/function. Further, it has shown potentially useful results in experimental diabetic complications. Similarly, a significant cardiovascular protective effect of catalpol was demonstrated, this being linked to reduced inflammation and ROS with improved cardiac function. Catalpol was tested in different experimental cancer models and was found to have a significant effect against cancers of the breast, stomach, lung, and colorectal cancer; one placebo-controlled clinical study demonstrated effectiveness against colorectal cancer. The anticancer effect appears to be due to reduced inflammation, apoptosis, angiogenesis, and arresting the cell cycle. Additionally, catalpol was shown to be effective against hepatotoxicity, asthma, and ovarian failure in experimental models. These biological effects of catalpol and their signal pathway connect with each other and influenced by each other. The common link in all these effects are the potentially linked to antioxidant and anti-inflammatory activity however, the real mechanism is still poorly understood. Pharmacokinetics studies of catalpol showed good oral bioavailability with shorter plasma half-life. Which suggest multiple dosing in a day is required for achieving steady state plasma concentration. This review discussed the biological effects and pharmacokinetics of catalpol in experimental animal models which is a potential drug candidate. Preclinical safety and toxicity studies of catalpol need to be conducted to understand its benefit to risk ratio. Similarly, only few clinical studies are reported yet, thus conducting clinical studies of catalpol remain an opportunity in future.

## Author Contributors

V.R., K.H.Y., and H.C. has contributed in writing. S.K.B. is responsible for conceptualizing, design, writing, formatting, and communicating the article. All authors have read and agreed to the published version of the manuscript.

## Figures and Tables

**Table 1:** Effect of catalpol in experimental models of inflammation and oxidative stress.

Experimental Model	Dose and Duration	Key Findings of Catalpol	References
Human aorta endothelial cells	Catalpol: 7.5, 15, or 30 µM for 24 h	Suppressed LDH secretion, MDA levels and the reduction in GSH Inhibited NF-κB activity and overproduction of ROS via the inhibition of Nox4 expression and endoplasmic reticulum stress Enhanced Bcl-2 and reduced Bax, Caspase-3, and Caspase-9 protein expression	[9]
BALB/c mice	Catalpol: 2.5, 5, or 10 mg/kg	Reduced levels of IL-6, IL-4, IL-1β, and TNF- α Up-regulated IL-10 expression Inhibited activation of NF-κB and MAPK signaling pathways	[11]
BALB/c mice	Catalpol: 5, or 10 mg/kg for 2 weeks	Inhibited ovalbumin-induced inflammation and IgE secretion in the lung as shown by decreases in IL-4 and IL-5 levels Inhibited aberrant eosinophil infiltration in the lungs and prevented the increase of eosinophil chemokine eotaxin and its receptor, CCR3	[13]
Ovalbumin induced asthma in BALB/c mice	5 mg/kg, i.p. 2 weeks	Significant decrease in inflammatory cells, eosinophil, IgE level in bronchoalveolar lavage fluid Marked reduction in IL-1, IL-4, and elevation in INF-γ in bronchoalveolar lavage fluid. Significant reduction in TLR-4 gene and protein expression in serum	[14]
Human THP-1 monocyte and A549 cell	Catalpol: 5, 10, 20, or 50 µM for 24 h	Reduced IL-1β and TNF- α levels Suppressed the activation of the downstream extracellular signal-reduced kinase and NF-κB inflammatory pathway	[15]
Human aorta epithelial cells	Catalpol: 10, 20, or 40 µM for 24 h	Inhibited oxidative stress and induced autophagy via activation of AMPK signaling pathway Increased SOD and suppressed Nox4 activity	[16]
Rat pheochromocytoma cells	Catalpol: 0.001, 0.01, 0.1, or 1 mM for 24 h	Suppressed the down-regulation of Bcl-2, up-regulation of Bax and release of mitochondrial cytochrome c to cytosol Alleviated Caspase-3 activation, PARP cleavage	[17]

**Table 2:** Effect of catalpol in experimental models of neurological disorders.

Experimental Model	Dose and Duration	Key Findings of Catalpol	References
Kunming mice	Catalpol: 5, 10, or 20 mg/kg, p.o., for 2 weeks Positive control: 10 mg/kg fluoxetine hydrochloride for 2 weeks	Reduced duration of immobility during tail suspension test and forced swim test Counteracted decrease in rectal temperature, akinesia and eyelid ptosis induced by reserpine Increased concentration of serotonin and its metabolite, 5-HIAA in brains of mice Significant antidepressant effect	[22]
Sprague-Dawley rats	Catalpol: 5, 10, or 20 mg/kg, for 5 weeks Positive control: 10 mg/kg fluoxetine hydrochloride for 2 weeks	Alleviated depressive-like behavior Decreased serum corticosterone levels Increased BDNF activity, mRNA expression of BDNF and TrkB Down-regulated mRNA expression of COX-2 PGE_2_ in the hippocampus and frontal cortex Improved cognitive ability	[23]
Kunming mice	Catalpol: 1, 3, or 9 mg/kg, for 3 days Positive control: 7.9 mg/kg edaravone or 105 mg/kg oxiracetam for 3 days	Induced neurological function recovery Enhanced spontaneous mobility and antidepressant effect Increased levels of ACh, ChAT, and BDNF in the hippocampus Neuroprotective effect against Alzheimer’s condition	[24]
CD-1 mice	Catalpol: 50 mg/kg, for 60 days	Improved memory deficits in neurodegenerative mice Elevated levels of muscarinic receptors, ChAT and BDNF in the brain of mice	[25]
Sprague-Dawley rats	Catalpol: 5 mg/kg, for 10 days	Increase in levels of GAP-43 and synaptophysin in the hippocampus Synaptophysin in the dentate granule layer of the hippocampus was increased Increase in protein kinase-C and BDNF in the hippocampus Increase in presynaptic proteins and up-regulation of relative signaling molecules indicates amelioration of neuroplasticity loss	[26]
Swedish mutant APP overexpressed N2a cells	Catalpol: 200 or 400 µ m for 18 h	Reduction in production of Aβ via non amyloidogenic APP pathway (enhanced expression of α-secretase, ADAM10)	[27]
C57BL/6 mice Ventral mesencephalons	Catalpol: 50 mg/kg, for 8 weeks Catalpol: 5, 15, or 50 mg/kg, for 8 weeks	Improved locomotor ability Elevated striatal dopamine levels Increased GDNF mRNA expression Protected the mesencephalic neurons from neurite shortening	[28]
Mesencephalic neurons	Catalpol: 0.05, 0.1, or 0.5 mM for 30 min	Increased neuron viability and reduced death of dopaminergic neurons Attenuated the loss of mitochondrial membrane potential Increase activity of antioxidant enzymes, glutathione peroxidase and superoxide dismutase Improved Parkinson’s disease condition	[29]

**Table 3:** Effect of catalpol in experimental models of diabetes mellitus and diabetes complications.

Experimental Model	Dose and Duration	Key Findings of Catalpol	References
Rats (STZ, 65 mg/kg)	0.1 mg/kg, i.v. injection, three days	Catalpol enhanced β-endorphin release from the isolated adrenal medulla of STZ-diabetic rats. Marked reduction of PEPCK expression in liver and an increased expression of GLUT 4 in skeletal muscle. Plasma glucose lowering action of catalpol failed to produce in opioid μ-receptor knockout mice. Catalpol increased glucose utilization through increase of β-endorphin secretion from adrenal gland in STZ-diabetic rats	[1]
Rats (STZ, 60 mg/kg)	0.01–0.1 mg/kg, i.v. injection	Significantly attenuated the increase of plasma glucose induced by an intravenous glucose challenge test in normal rats. Catalpol enhanced the uptake of radioactive glucose in the isolated soleus muscle of diabetic rat in a concentration-related manner Increase glycogen synthesis in liver of diabetic rats	[46]
Male Sprague–Dawley rats (STZ, 65 mg/kg, i.p.)	10, 50, and 100 mg/kg, intra-gastric infusion, Six (6) weeks	Catalpol (50 and 100 mg/kg) showed significant and dose dependent reduction in blood glucose Significant improvement in CAT, SOD, and GSH-PX and attenuation of MDA in hippocampus was observed. NGF level in hippocampus was significantly and dose dependently increased with all the doses of catalpol. Neural density in CA1 region of hippocampus is significantly increased whereas, the density of TUNNEL positive cells was significantly decreased. Significantly improved the cognitive function of diabetic rats in Y-type maze	[47]
STZ-induced diabetes rats with balloon-injured carotid arteries		Neointimal hyperplasia area was reduced in carotid arteries. Reduced levels of monocyte chemoattractant protein-1 in carotid arteries	[48]
Male C57BL/6 mice (STZ, 180 mg/kg)	10 mg/kg/d, i.p. 14 days	Reduction in 24 h urinary protein excretion, serum creatinine levels, and blood urea nitrogen. Significantly attenuated the elevated Grb10, caspase-3 expression in diabetic kidneys. Significantly higher in IGF-1 mRNA levels and IGF-1R phosphorylation (tyr1161) in the kidneys of catalpol-treated diabetic mice. Pathological severity and kidney fibrosis is significantly attenuated. Improved the diabetes-associated impaired renal functions and ameliorate pathological changes in diabetic kidneys	[49]
C57BL/6 J male mice (HFD/STZ, 85 mg/kg, i.p.)	50, 100, or 200 mg/kg, p.o.; 4 weeks	Significant and dose dependent reduction of FBG, AUC for intraperitoneal glucose tolerance test (IPGTT-AUC). Catalpol (200 mg/kg) reduced TG (30%) and TC (45%). Skeletal muscle mitochondrial ATP content was significantly increased by approx 3.0 and 2.5 folds after the administration of 200 and 100 mg/kg catalpol. Mitochondrial membrane potential of isolated mitochondria in skeletal muscle was improved in a dose dependent manner after catalpol treatment. Significant and dose dependent increase in the mtDNA copy number in skeletal muscle. Catalpol (200 mg/kg) rescued muscle mitochondrial injury. PGC1α mRNA level was increased by 1.2 folds in the skeletal muscle of mice treated with catalpol (200 mg/kg)	[50]
db/db mice	200 mg/kg, p.o. 8 weeks	Increased phosphorylation of IRS-1 and Akt, increased PI3K and GLUT4 levels in skeletal muscle. Increased insulin sensitivity and glucose Uptake in skeletal muscle Enhanced MyoD/MyoG-mediated myogenesis in skeletal muscle cells. No effect on insulin secretion	[51]
C2C12 cells	10, 30, or 100 μM		
db/db mice	40, 80, 160 mg/kg, p.o. 4 weeks	Catalpol (80 and 160 mg/kg) significantly decreased the fasting blood glucose, glycated serum protein, total cholesterol and triglycerides concentrations Catalpol (80 and 160 mg/kg) exhibited a higher glucose tolerance and improve insulin resistance. Catalpol (80 and 160 mg/kg) significantly increased protein expression of p-AMPKα1/2 in liver, adipose tissue and skeletal muscle; GLUT-4 in skeletal muscle and adipose tissues; Catalpol (80 and 160 mg/kg) significantly decreased ACC and HMGCR mRNA expressions in liver It improved glucose tolerance, ameliorated insulin resistance, inhibits the triglyceride and cholesterol synthesis	[52]
C57BL/6 J mice (HFD, 60% calorie with STZ, 40 mg/kg, for five consecutive days)	100 and 200 mg/kg/d, p.o.; 4 weeks	Increased GSK-3β phosphorylation at Ser9 and decreased GS phosphorylation at Ser641 in glucosamine-induced HepG2 cells and in the livers of HFD/STZ mice. Increased FOXO1 phosphorylation at Ser256 in glucosamine-induced HepG2cells, and inhibited PEPCK and G6pase expressions in glucosamine-induced HepG2 cells and in the livers of HFD/STZ mice. Decreased IRS-1 phosphorylation at Ser307in the livers of HFD/STZ mice, and increased Akt phosphorylation at Ser473 in glucosamine-induced HepG2 cells and in livers of HFD/STZ mice. Increased AMPK phosphorylation at Thr172 in glucosamine-induced HepG2 cells and in the livers of HFD/STZ mice. Reduced MDA level and increased GSH and SOD levels in the serum of HFD/STZ-induced mice and glucosamine-induced HepG2 cells. Reduced hepatic gluconeogenesis may ascribe to PI3K/Akt/FOXO1-mediated PEPCK and G6pase inhibition, and increased hepatic glycogen synthesis may attribute to PI3K/Akt/GSK3β mediated GS activation	[53]
glucosamine-induced HepG2 cells	20, 40, and 80 µM catalpol		
C57BL/6 mice (HFD, 45% calorie)	100 mg/kg, p.o. 4 weeks	FBG levels, FPI levels, HOMA-IR, and AUC of OGTT were significantly reduced in the catalpol treated group. Reduction in adipose tissue inflammation, gene expression of the general macrophage marker F4/80 was markedly attenuated in epididymal adipose tissue. Pro-inflammatory genes such as TNF-α, IL-6, IL-1b, MCP-1, iNOS, and CD11c were markedly reduced by catalpol in adipose tissue. Anti-inflammatory genes such as arginase 1, Ym-1, IL-10, MGL1, Clec7a, and MMR were significantly increased by catalpol in adipose tissue. Significant decrease the phosphorylation of JNK and IKKβ, and decreased NF-kB p50 activation in adipose tissue	[54]
Rats with initial high-fat and high-sugar diet (3 weeks), followed by STZ (30 mg/kg, i.p.) for 3 days	5, 10, 20, or 50 mg/kg, i.v. 2 weeks	Significant plasma glucose-lowering activities (65.8 ± 3.07%) at catalpol (50 mg/kg). Catalpol (50 mg/kg) showed significant attenuation of TC, TG and increase in HDL-C. Catalpol (50 mg/kg) significantly increased the SOD, GSH-PX, and CAT and reduced the MDA level in plasma. Catalpol reduced the damage to the pancreas in STZ-fat-diabetic rats	[55]
Male C57/BL6N (HFD and low dose STZ (50 m/kg, i.p.)	200 mg/kg, p.o. 4 weeks	Significant reduction in FBG, HOMA_IR, plasma and liver triglyceride. Histologically, structural integrity of hepatocyte and glycogen reserve was partially restored by catalpol in diabetic liver. Glucokinase and PPAR-γ gene and PPAR-γ protein was upregulated in liver of catalpol treated group	[56]
Rats (HFD/STZ)	10, 50, 100 mg/kg, Six (6) weeks	Endothelial damage of thoracic aorta was attenuated significantly ROS level of thoracic aorta and serum level of 8-iso-PGF2α were decreased significantly Serum NO and SOD levels were remarkably elevated Expression of Nox4, p22phox mRNA, and protein in thoracic aorta were significantly reduced Catalpol has protective effect on endothelial of T2DM which may be associated with the down-regulation of Nox4 and p22phox expression, inhibiting oxidative stress	[57]
Male Sprague-Dawley (SD) rats (HFD/STZ, 35 mg/kg, i.p.)	30, 60, and 120 mg/kg, p.o. 10 weeks	Catalpol (60 and 120 mg/kg) significantly decreased the RBG, GSP and BUN but no difference in plasma creatinine level. Significantly reduced Ang II, TGF-β1, CTGF, FN, and Col IV concentrations in renal cortex. Reduced the mRNA expression of TGF-β1 and CTGF in renal cortex. Reduced the kidney hypertrophy, kidney weight index and microscopic damage of glomeruli	[58]
C57BLKS/J db/db mice	Chow diet supplemented with catalpol (1 g/kg), Sixteen (16) weeks	Significant reduction of body weight in catalpol treated group from 8 weeks of catalpol treatment. Significant reduction of water consumption in catalpol treated group from 12 weeks onwards of catalpol consumption. Fasting blood glucose levels in the catalpol group were significantly lower than that of the model group from the 4th to 16th week catalpol treatment. Insig1, Scd2, and Slc5a8 genes in kidney was restored by catalpol	[59]

**Table 4:** Effect of catalpol in experimental models of cardiovascular disorders.

Experimental Model	Dose and Duration	Key Findings of Catalpol	References
Adult male rats (30 min of myocardial ischemia and 3 h of reperfusion)	5 mg/kg, i.p., 5 min before reperfusion	Increased Akt and eNOS phosphorylation, NO production, anti-oxidant capacity and reduced MI/R-induced iNOS expression and superoxide anion production in I/R hearts Peroxynitrite radical formation was markedly reduced in myocardial tissue. Significantly improved cardiac functions, reduced myocardial infarction, apoptosis and necrosis of cardiomyocytes after Myocardial ischemia/reperfusion (MI/R) injury	[66]
Isoproterenol (ISO)-induced myocardial infarction (MI) ISO (85 mg/kg, s.c. 2 days)	10 mg/kg, i.p. 10 days	Significant protection against myocardial infarction. Attenuated serum LDH and CK-MB level. Increased myocardial SOD and MDA level. Reduced the expression of inflammatory markers (TNF-α and IL-1β) genes and proteins in myocardial tissue.	[67]
Isoproterenol (ISO)-induced myocardial infarction (MI) ISO (85 mg/kg, s.c. 2 days)	5 and 10 mg/kg/day, i.p. 10 days	Catalpol pretreatment (10 mg/kg) attenuated the dropping of SBP, DBP, and MBP in ISO treated MI rats. Catalpol (5 and 10 mg/kg) showed improvement in +LVdp/dt_max_ LVEDP. Catalpol (5 and 10 mg/kg) showed higher plasma apelin levels whereas, catalpol (10 mg/kg) significantly increased myocardium apelin and APJ expression than the ISO group. Catalpol pretreatment (5 and 10 mg/kg) reduced the rate of cell apoptosis in the left ventricular tissues Significant reduction in Bax expression and increased Bcl-2 expression in the left ventricular tissues Significant decrease in the caspase-3 and caspase-9 activity in myocardial tissue	[68]
Isoprenaline (10 mg/kg, s.c) induced MI in SD rats	10, 20, 40 mg/kg, p.o. Three (3) weeks	Catalpol reduced S-T elevation in MI rats in ECG recording. Plasma SOD level is increased whereas MDA, LDH, and CK level decreased. These effects are marked in 20, and 40 mg/kg. The peripheral blood Endothelial progenitor cells (CD34+/CD133+/VEGFR2+) count was increased in catalpol treatment (0.51%, 0.97%, and 3.22% respectively with 10, 20, and 40 mg/kg of catalpol). Notch1 and Jagged1 protein expression in myocardial tissue was significantly increased after catalpol treatment. Inflammatory cell infiltration and myocardial tissue damage was attenuated by catalpol	[69]
H9c2 embryonic rat cardiac cells	0.1, 1, and 10 mg/mL 24 h	Marked elevation in autophagy-related proteins (Beclin1, LC3, and ULK) in catalpol-treated cardiac cells. Significant anti-apoptotic and antioxidative effect of catalpol was observed and linked to autophagy. Anti-apoptotic and anti-oxidant effect of catalpol was completely abashed in presence of autophagy inhibitor 3-Methyladenine. Marked elevation in mitophagic related proteins (ATG5, Beclin 1, LC3, p62). Tamoxifen, an estrogen receptor inhibitor completely blocked the upregulation of catalpol induced mitophagic related proteins	[70]
High-cholesterol fed diet to male New Zealand White rabbits	5 mg/kg/day, 12 weeks	Remarkable reduction of TC, TG, and LDL-C in serum. Whereas, HDL-C was elevated. Significant reduction in the level of TNF-α, IL-6, MCP-1, soluble VCAM-1, and soluble ICAM-1 in the serum. Significant reduction in VCAM-1, MCP-1, TNF-α protein, iNOS, MMP-9, and NF-κB protein65 in the aortic arch Significant anti-atherosclerosis effect	[71]
Middle cerebral artery occlusion in Rats	5 mg/kg, i.p. 7 days	Catalpol improved behavioral impairment, cerebral blood flow in rats after cerebral ischemia. Angiogenesis was increased based on the increased expression of von Willebrand Factor and proliferating cell nuclear antigen expression. EPO, EPOR, JAK2, p-JAK2, STAT3, p-STAT3, and VEGF protein expression in the brain tissue was significantly improved. Catalpol increased angiogenesis after stroke and it could be due to activation of JAK2-STAT3 signaling pathway	[72]
Hydrogen peroxide (H_2_O_2_) induced apoptosis in Human umbilical vein endothelial cells (HUVECs)	0.1, 1, and 10 μg/mL, 48 h	Significantly and dose dependently reduced H_2_O_2_- induced intracellular ROS release. Increased the Bcl-2 expression, Akt activation and Bad phosphorylation. Significantly decrease the Bax expression. Significant anti-apoptotic effect	[73]

**Table 5:** Effect of catalpol in experimental models of cancer.

Experimental Model	Dose and Duration	Key Findings of Catalpol	References
Human gastric cancer cells (MKN-45) Athymic nude mice	Catalpol (in vitro): 2.5, 5, 10, 20, 40, 80, or 160 µM for 24 h Catalpol (in vivo): 10, 20, or 40 mg/kg, for 3 weeks	Reduced proliferation and migration of cancer cells (as shown by suppression of MMP-2, α-SMA, RhoA, ROCK1, and N-cadherin) Induced apoptosis in cancer cells (as shown by elevation of apoptosis-associated markers, cleaved Caspase-3 and PARP Prevented tumor growth in xenograft nude mice	[75]
Human solid tumor cell lines (A2780, HBL-100, HeLa, SW1573, T-47D and WiDr)	Catalpol: 1, 2, 3, or 5 µm for 24 h	Showed antiproliferative activity Cell cycle arrest at G1 phase Reduced expression of Cyclin D1	[76]
Human non–small-cell lung cancer (NSCLC) cells- A549 cells		Significantly inhibited the TGF-β1-induced cell migration and invasion of A549 cells. Attenuated MMP-2 and MMP-9 expression. Significant attenuation of Smad2/3 activation and NF-κB signaling pathways induced by TGF-β1 in A549 cells	[77]
Human colorectal cancer cells (HCT116)	Catalpol: 0, 25, 50, or 100 µg/mL for 48 h	Inhibited HCT116 cancer cell proliferation via the downregulation of the PI3K-Akt signaling pathway Induced apoptosis of HCT116 cancer cells via increased activities of caspase-3 and caspase-9, and upregulation of microRNA-200 expression	[78]
Colon cancer cells (CT26) C57BL6 mice and SD rats	Catalpol: 2.5, 5, 10, 20, 40, or 80 µM for 24 and 48 h	Inhibited proliferation and growth of CT26 cancer cells in vitro and in vivo Suppressed tumor cell-induced vascularization of endothelial cells and rat aortic ring angiogenesis Reduced expression of angiogenic markers, VEGF, VEGFR2, bFGF and HIF-1α in colon cancer tissues Halted the expression of pro-inflammatory factors, IL-1β, IL-6, IL-8, COX-2, and iNOS	[79]
Randomized, placebo-controlled parallel clinical study in patients that had undergone surgical resection for locally advanced colon adenocarcinoma (n = 345)	Catalpol: 10 mg/kg twice daily for 12 weeks Positive control: 5 mg/kg bevacizumab twice weekly for 12 weeks Placebo group: no treatment	Reduced serum levels of CA 19-9, CEA, MMP-2, and MMP-9 (colon cancer biomarkers) compared to the placebo group Reduced tumor recurrences and improvement in overall survival compared to the placebo and positive control group	[80]
Human breast cancer cells (MCF-7)	Catalpol: 0, 25, 50, or 100 µg/mL for 24, 48 and 72 h	Reduced proliferation and induced apoptosis in MCF-7 cancer cells Reduced expression of the tumor invasion enzyme, MMP-16 Upregulated expression of microRNA-146a (reducing metastatic potential)	[81]
Human osteosarcoma cancer cells (MG63 and U2OS)	Catalpol: 20, 40, or 80 µm for 48 h	Reduced progression, viability and migration of osteosarcoma cells Decreased expression of RACK1 and MMP-2 indicating cancer cell migration inhibition Induced apoptosis in cancer cells as shown by improved cleavage of Caspase-8, Caspase-3, Caspase-9 and PARP	[82]

**Table 6:** Other biological effects of catalpol and their underlying mechanism.

Experimental Model	Dose and Duration	Key Findings of Catalpol	References
Lipopolysaccharide (50 μg/kg, i.p.)/d-galactosamine (800 mg/kg, i.p.) -induced acute liver injury in mice	2.5, 5, 10 mg/kg, i.p. Three (3) days	Catalpol attenuated serum ALT and AST level and exhibited hepatoprotective effect Catalpol ameliorated hepatic tissue pathological changes (hemorrhagic necrosis, destruction of hepatic architecture, massive infiltration of inflammatory cells). Catalpol decreases MDA production, MPO, TNF-α production NF-κB activation. Catalpol dose dependently decreased the NF-κB p65 and IκBα phosphorylation and increased the expression of Nrf2 and HO-1	[97]
Triptolide (TP) induced hepatotoxicity in Human normal hepatocytes (L-02 cells)	2, 10, 50, and 250 μg/mL	Catalpol significantly reduced TP-induced inhibition of nuclear factor erythroid-2-related factor-2 (Nrf2) transcription. Catalpol (10 μg/mL) was showed best protective activity against TP-induced hepatocyte injury. Catalpol significantly reduce ALT and AST leakage due to TP-induced hepatocyte injury. Catalpol (10 μg/mL) increased HO-1 and NQO1, Nrf2 protein expression. Nrf2/ARE pathway activation Catalpol exhibited significant hepatoprotective effect	[98]
Hepatic stellate cells (HSCs) SD rats	Catalpol (in vitro): 0.625, 1.25, 2.5, 5, 10, 20, or 40 µM for 24 h Catalpol (in vivo): 10, 20, or 40 mg/kg for 4 weeks	Protected liver from CCl_4_-induced damage by mitigating hepatic steatosis, necrosis and fibrotic septa Down-regulated pro-inflammatory cytokines, IL-1β, TNF- α, IL-18, IL6, and COX-2	[99]
14 months old SD female rats (ageing model)	1, 3, and 5 mg/kg, p.o. Four (4) weeks	Prevented the ovary index (g/100 g body weight) loss. Catalpol normalized the ovarian ultrastructure at 3 mg/kg. Catalpol (3 and 5 mg/kg) reduced the apoptosis of ovarian granule cells. Catalpol significantly increased the levels of estradiol and progesterone and reduced the levels of FSH and LH. Catalpol significantly protected ovarian failure	[100]

**Table 7:** Pharmacokinetics of catalpol in different studies.

Sample	Rat Plasma	Rat Plasma	Rat Plasma	Rat Plasma	Rat CSF
Dose	50 mg/kg, p.o.	6 mg/kg, i.v.	10 mg/kg, i.v.	8 mg/kg, p.o.	6 mg/kg, i.v.
Tmax (h)	1.333 ± 0.408	-	-	2.8 ± 0.837	0.08 ± 0.02
Cmax (ng/mL)	23,318 ± 10,468	23 617.4 ± 914.7	-	1680 ± 120	675.9 ± 198.4
T_1/2_ (h)	1.212 ± 0.388	0.71 ± 0.23	0.984 ± 0.229	3.275 ± 1.192	1.52 ± 0.74
AUC _(0–t)_	-	11 432.3 ± 1582.5 ng·h/mL	5951.125 ± 1247.247 µg·h/L	584.80 ± 107.29 µg·min/mL	594.5 ± 81.3 ng·h/mL
AUC _(0–∞)_	69,520 ± 22,927 ng·h/mL	11 532.9 ± 1643.0 ng·h/mL	5954.076 ± 1248.205 µg·h/L	666.30 ± 194.60 µg·min/mL	671.5 ± 109.1 ng·h/mL
MRT _(0–∞)_ (h)	3.273 ± 0.365	0.70 ± 0.20	0.0454 ± 0.140	-	2.12 ± 1.0
V (L/kg)	1.428 ± 0.681	-	0.348 ± 0.075	-	-
CL (L/h/kg)	0.824 ± 0.317	-	0.348 ± 0.075	0.9 ± 0.24	-
Method	LC/MS/MS	HPLC–APCI–MS/MS	LC–ESI-MS/MS	UPLC–MS	HPLC–APCI–MS/MS
Reference	[107]	[108]	[105]	[109]	[108]

## Abbreviations

±LVdp/dt_max_	Left ventricular maximum rate of positive or negative pressure development
5-HIAA	5-hydroxyindoleacetic acid
5-HT	5-hydroxy tryptophan
A2780	Human ovarian cancer cell line
A549	Adenocarcinomic human alveolar basal epithelial cell line
ACC	Acetyl-CoA carboxylase
Ach	Acetylcholine
AD	Alzheimer’s disease
ADAM10	A Disintegrin and Metalloproteinase 10
AGE	Advanced glycation end product
Akt	Protein kinase B
ALT	Alanine aminotransferase
AMPK	Adenosine Monophosphate Activated Protein Kinase
Ang II	Angiotensin II
APJ	Apelin receptor
APP	Amyloid precursor protein
AST	Aspartate transaminase
ATG5	Autophagy-related gene 5
ATP	Adenosine triphosphate
AUC	Area under the curve
Aβ	Amyloid β
Bax	BCL2-associated X protein
Bcl-2	B-cell lymphoma 2
BDNF	Brain-derived neurotrophic factor
bFGF	Basic fibroblast growth factor
BUN	Blood urea nitrogen
CA 19-9	Cancer antigen 19-9
CA1	Carbonic anhydrase 1
CAT	Catalase
CCL4	Carbon tetrachloride
CCR3	CC chemokine receptor 3
CD-1	Cluster of differentiation 1
CD133+	Cluster of differentiation 1
CD34+	Cluster of differentiation 1
CEA	Carcinoembryonic antigen
ChAT	Choline acetyltransferase
CK-MB	Creatine Kinase-MB
Clec7a	C-type lectin domain family 7-member A
Col IV	Collagen type IV
COX-2	Cyclooxygenase-2
CT26	Colorectal cancer cell line
CTGF	Connective tissue growth factor
CXCR4	Chemokine receptor 4
DBP	Diastolic blood pressure
dNTP	Deoxynucleoside triphosphates
EMT	Epithelial mesenchymal transition
eNOS	Endothelial nitric oxide synthase
EPO	Erythropoietin
EPOR	Erythropoietin receptors
FBG	Fasting blood glucose
FN	Fibronectin
FOXO1	Fork head box protein O1
FPI	Fasting plasma insulin
FSH	Follicle-stimulating hormone
FST	Forced swim test
G6pase	Glucose-6 phosphatase
GAP-43	Growth-associated protein 43
GDNF	Glial cell-derived neurotrophic factor
GLUT4	Glucose transporter 4
Grb10	Growth factor receptor-bound protein 10
GS	Glycogen synthase
GSH-PX	glutathione peroxidase
GSK-3β	Glycogen synthase kinase beta
GSP	Glycated serum protein
H_2_O_2_	Hydrogen peroxide
HBL-100	Human breast mammary gland cell line
HCT116	Human colorectal carcinoma cell line
HDL-C	High-density lipoprotein cholesterol
HFD	High fat diet
HIF-1α	Hypoxia-inducible factor 1α
HMGCR	Hydroxymethyl glutaric acid acyl CoA reductase
HO-1	Haem oxygenase-1
HOMA-IR	Homeostatic model assessment of insulin resistance
HPA	Hypothalamic-pituitary-adrenal
HSCs	Hepatic stellate cells
HUVECs	Human umbilical vein endothelial cells
HUVECs	Human umbilical vein endothelial cells
ICAM-1	Intercellular adhesion molecule-1
IDO	Indoleamine 2,3- dioxygenase
IgE	Immunoglobulin E
IGF-1	Insulin-like growth factor 1
IGF-1R	Insulin-like growth factor 1 receptor
IKKβ	Inhibitor of nuclear factor kappa-B kinase subunit beta
IL-10	Interleukin-10
IL-1β	Interleukin-1β
IL-4	Interleukin-4
IL-5	Interleukin-5
IL-6	Interleukin-6
IL-8	Interleukin-8
iNOS	Inducible nitric oxide synthase
Insig1	Insulin-induced gene 1
IPGTT	Intraperitoneal glucose tolerance test
IRS-1	Insulin receptor substrate 1
ISO	Isoproterenol
IκBα	Inhibitor of kappa B alpha
JAK2	Janus kinase2
JNK	c-Jun N-terminal kinases
KWI	Kidney weight index
LC3	Microtubule-associated proteins 1A/1B light chain 3B
LDH	Lactate dehydrogenase
LDL-C	Low density lipoprotein cholesterol
LH	Luteinizing hormone
LPS	Lipopolysaccharide
LVEDP	Left ventricular end diastolic pressure
LVESP	Left ventricular end-systolic pressure
LY294002	2-(4-morpholinyl)-8-phenyl-4H-1-benzopyran- 4-one
MAPK	Mitogen activated protein kinase
MBP	Mean blood pressure
MCF-7	Human breast adenocarcinoma cell line
MCP-1	Monocyte chemoattractant protein 1
MDA	Malondialdehyde
MG63	Human osteosarcoma cell line
MGL1	Macrophage galactose-type lectin 1
MI/R	Myocardial ischemia/reperfusion
MI	Myocardial infarction
MMP-16	Matrix metalloproteinases-16
MMP-2	Matrix metalloproteinases-2
MMP-9	Matrix metalloproteinases-9
MMR	Measles mumps and rubella vaccine
MPO	Myeloperoxidase
MPTP	1-methyl-4-phenyl-1,2,3,4-tetrahydropyridine
mRNA	Messenger ribonucleic acid
mtDNA	Mitochondrial deoxyribonucleic acid
MyoD	Myoblast determination protein
MyoG	Myogenin
N2a	Neuroblastoma 2a cell line
NE	Norepinephrine
NF-κB	Nuclear factor-κB
NGF	Nerve growth factor
NO	Nitric oxide
Nox4	NADPH oxidase 4
NQO1	NADPH quinone 1
NRF-1	Nuclear respiratory factor 1
NSCLC	Non-small cell lung cancer
OGTT	Oral glucose tolerance test
p-AMPK	Phosphorylated Adenosine Monophosphate Activated Protein Kinase
PARP	Poly-ADP ribose polymerase
PC12	Pheochromocytoma cell line
PDGF	Platelet derived growth factor
PEPCK	Phosphoenolpyruvate carboxykinase
PGC1α	Peroxisome proliferator-activated receptor gamma coactivator 1α
PGE_2_	Prostaglandin E2
PGF2α	Prostaglandin F2 alpha
PI3K	Phosphoinositide 3-kinase
PMA	Phorbol-12-myristate-13-acetate
RACK1	Receptor of activated protein C kinase 1
RBG	Random blood glucose
RhoA	Ras homolog gene family member A
ROCK1	Rho associated coiled-coil containing protein kinase 1
ROS	Reactive oxygen species
SBP	Systolic blood pressure
Scd2	Steaoryl-coenzyme A desaturase 2
SD	Sprague Dawley
SiRNA	Short interfering ribonucleic acid
Sirt1	Sirtuin 1
SOD	Superoxide dismutase
STAT3	Signal transducer and activator of transcription 3
STZ	Streptozotocin
T2DM	Type 2 diabetes mellitus
T-47D	Human breast cancer cell line
TC	Total cholesterol
TG	Triglycerides
TGF-β1	Transforming growth factor-β1
TH	Tyrosine hydroxylase
THP-1	Human leukemia monocytic cell line
TNF-α	Tumor necrosis factor-α
TP	Triptolide
TrkB	Tropomyosin receptor kinase B
TST	Tail suspension test
TUNEL	Terminal deoxynucleotidyl transferase dUTP Nick-End labelling
U2OS	Human osteosarcoma cell line
ULK	Unc-51-like kinases
UPE	Urinary protein excretion
VCAM-1	Vascular cell adhesion molecule-1
VEGF	Vascular endothelial growth factor
VEGFR2	Vascular endothelial growth factor receptor 2
WiDr	Colon adenocarcinoma cell line
α-SMA	Alpha smooth muscle actin
